# Macrophage responses to lipopolysaccharide are modulated by a feedback loop involving prostaglandin E_2_, dual specificity phosphatase 1 and tristetraprolin

**DOI:** 10.1038/s41598-017-04100-1

**Published:** 2017-06-28

**Authors:** Tina Tang, Thomas E. Scambler, Tim Smallie, Helen E. Cunliffe, Ewan A. Ross, Dalya R. Rosner, John D. O’Neil, Andrew R. Clark

**Affiliations:** Institute of Inflammation and Ageing, College of Medical and Dental Sciences, University of Birmingham, Birmingham, B15 2WB UK

## Abstract

In many different cell types, pro-inflammatory agonists induce the expression of cyclooxygenase 2 (COX-2), an enzyme that catalyzes rate-limiting steps in the conversion of arachidonic acid to a variety of lipid signaling molecules, including prostaglandin E_2_ (PGE_2_). PGE_2_ has key roles in many early inflammatory events, such as the changes of vascular function that promote or facilitate leukocyte recruitment to sites of inflammation. Depending on context, it also exerts many important anti-inflammatory effects, for example increasing the expression of the anti-inflammatory cytokine interleukin 10 (IL-10), and decreasing that of the pro-inflammatory cytokine tumor necrosis factor (TNF). The tight control of both biosynthesis of, and cellular responses to, PGE_2_ are critical for the precise orchestration of the initiation and resolution of inflammatory responses. Here we describe evidence of a negative feedback loop, in which PGE_2_ augments the expression of dual specificity phosphatase 1, impairs the activity of mitogen-activated protein kinase p38, increases the activity of the mRNA-destabilizing factor tristetraprolin, and thereby inhibits the expression of COX-2. The same feedback mechanism contributes to PGE_2_-mediated suppression of TNF release. Engagement of the DUSP1-TTP regulatory axis by PGE_2_ is likely to contribute to the switch between initiation and resolution phases of inflammation.

## Introduction

The cyclooxygenase enzymes COX-1 and COX-2, encoded by the genes *Ptgs1* and *Ptgs2*, catalyze rate-limiting steps in the synthesis of various prostanoid signaling molecules from the lipid precursor arachidonic acid^1, 2^. COX-1 is constitutively expressed by many cells. In contrast, COX-2 is expressed at low levels by the majority of cells, but transiently induced in response to growth factors, stresses or pro-inflammatory stimuli. Prostaglandin E_2_ (PGE_2_) is the major downstream product of COX-2-mediated arachidonic acid metabolism in many cells. PGE_2_ increases blood flow, vascular permeability and nociception, thereby contributing to all four of the cardinal signs of inflammation; redness, swelling, heat and pain. The pro-inflammatory actions of PGE_2_ underlie the clinical usefulness of non-steroidal anti-inflammatory drugs (NSAIDs), which inhibit both COX-1 and COX-2^2^. However, constitutive COX-1-mediated prostaglandin synthesis in the gastric mucosa helps to maintain the integrity of this vulnerable tissue^1^, which accounts for the increased incidence of gastric ulcers amongst patients using NSAIDs for prolonged periods. The rationale for the generation of COX-2-selective inhibitors was based on the assumption that COX-1 functions principally as a homeostatic enzyme, whereas COX-2 functions principally as a pro-inflammatory mediator. Selective inhibitors of COX-2 were predicted to exert anti-inflammatory effects whilst sparing gastric side effects^3^.

As we are reminded by the costly failure of the COX-2-selective inhibitor Rofecoxib (Vioxx), biology is rarely so straightforward or convenient^4^. COX-1-dependent synthesis of thromboxane by platelets promotes vasoconstriction and platelet aggregation. These prothrombotic actions are opposed by prostaglandin I_2_ (prostacyclin). The significantly increased risk of myocardial infarction and stroke in patients taking Vioxx eventually led to the withdrawal of this drug from the market. The basis of elevated cardiovascular risk is still not fully understood, but has been ascribed to an imbalance between COX-1-mediated pro-thrombotic and COX-2 mediated anti-thrombotic influences. Another confounding aspect of prostaglandin biology is that the actions of PGE_2_ are not invariably pro-inflammatory; nor are the effects of COX-2-selective inhibitors invariably anti-inflammatory. PGE_2_ has been shown to enhance expression of IL-10, inhibit the expression of TNF and other inflammatory mediators, and promote the differentiation of macrophages towards an alternatively-activated, anti-inflammatory M2 phenotype^5–10^. In rheumatoid synovial explant cultures or peripheral blood-derived monocytes, NSAIDs increased the expression of TNF^11^. Prior *in vivo* exposure to COX-2-selective inhibitors also primed human peripheral blood monocytes and mouse peritoneal macrophages for increased expression of TNF in response to an LPS challenge^11, 12^.

Cellular responses to PGE_2_ are mediated by four G protein-coupled receptors named EP1-EP4, which are the products of the genes *Ptger1*-*Ptger4* ^13, 14^. These receptors differ in their affinity for PGE_2_ and in the signal transduction pathways that they engage. Both EP2 and EP4 are Gα_s_-linked and activate adenylyl cyclase to elevate intracellular cAMP levels. EP4 has additionally been shown to signal via PI3K. EP1, which is coupled to Gα_q_, signals via phospholipase C to induce calcium flux. EP3 exists in a number of distinct forms arising from differential splicing of the *Ptger3* transcript, and appears to be promiscuous in terms of its signaling pathway engagement. There is clear potential for cell-specific programing of responses to PGE_2_ via modulation of the expression of the four receptors or their variants. Anti-inflammatory actions of PGE_2_ have generally been ascribed to EP4 and/or EP2^5, 6, 15–17^, however molecular mechanisms remain unclear. The increased expression of the anti-inflammatory cytokine IL-10 does not provide an explanation, as PGE_2_ can still inhibit macrophage expression of TNF in the absence of IL-10^18, 19^.

The inflammation-induced biosynthesis of PGE_2_ is regulated largely at the level of *Ptgs2* gene expression. The typical transient pattern of expression of *Ptgs2* mRNA is only partly explained by transcriptional activation involving nuclear factor κB (NF-κB) and other transcription factors^20^. Efficient expression also requires the stabilization of *Ptgs2* mRNA via the mitogen-activated protein kinase (MAPK) p38 signaling pathway, and conversely, MAPK p38 inhibitors accelerate the degradation of *Ptgs2* mRNA^21, 22^. This post-transcriptional regulation is mediated by an adenosine/uridine-rich element (ARE) immediately 3′ to the *Ptgs2* translation termination codon. When inserted into a stable reporter mRNA, the *Ptgs2* ARE confers rapid decay that is mediated by shortening of the protective poly-(A) tail (deadenylation), and can be prevented by activation of MAPK p38^23–25^. This sequence element is therefore similar to MAPK p38-responsive mRNA destabilizing elements present in pro-inflammatory mRNAs such as *Tnf*, *Csf2*, *Cxcl1*, *Il6* and many others^26^.

The mouse *Zfp36* gene enodes the ARE-binding protein tristetraprolin (TTP)^27, 28^. In *Zfp36*−/− macrophages lacking TTP protein, *Ptgs2* mRNA was highly stable and could not be destabilized by a MAPK p38 inhibitor^29^. TTP binds to AREs in the 3′ untranslated regions of target transcripts and recruits a complex of deadenylase enzymes, which catalyzes shortening of the poly-(A) tail, usually as a prelude to the rapid destruction of the mRNA body. The mRNA-destabilizing activity of TTP is regulated by a phosphorylation switch^30^. Pro-inflammatory agonists and cell stresses activate MAPK p38, which in turn phosphorylates and activates MK2 (MAPK-activated protein kinase 2). MK2 phosphorylates serines 52 and 178 of TTP, resulting in the recruitment of 14–3–3 adaptor proteins, impairment of the interaction between TTP and the deadenylase complex, and consequent stabilization of target mRNAs^31–33^. Protein phosphatase 2A (PP2A) catalyzes the removal of these two phosphate groups and the activation of TTP. Therefore a dynamic equilibrium exists between forms of TTP that are phosphorylated or unphosphorylated at serines 52 and 178, and this equilibrium favors the stabilization of TTP-regulated mRNAs under conditions of strong MAPK p38 activity. Coupling between MAPK p38 activity and the stability of pro-inflammatory mRNAs contributes to the precise orchestration of the on and off phases of inflammatory responses^34–36^.

The activity of MAPK p38, and hence the phosphorylation state of TTP, is regulated by a negative feedback loop involving dual specificity phosphatase 1 (DUSP1)^37^. Pro-inflammatory stimuli induce MAPK p38-dependent expression of DUSP1, which then dephosphorylates and inactivates MAPK p38 to enforce the off-phase of the inflammatory response. Due to the failure of this feedback mechanism, *Dusp1*−/− mice and cells are prone to respond excessively to pro-inflammatory agonists, and over-express many inflammatory mediators, including COX-2^38, 39^. The over-expression of COX-2 by *Dusp1*−/− macrophages is not fully understood; both transcriptional and post-transcriptional mechanisms have been proposed^40, 41^. Here, we investigate the functional relationship between DUSP1, MAPK p38, TTP and COX-2. First, we use a number of genetically modified mouse strains to demonstrate that DUSP1 and MAPK p38 control the expression of COX-2 by regulating the phosphorylation of TTP at serines 52 and 178. Next we turn to the effect on macrophages of the major COX-2 product, PGE_2_. We show that PGE_2_ enhances the expression of DUSP1 and thereby downregulates the expression of COX-2, creating another auto-regulatory feedback mechanism. PGE_2_ also acts via the receptor EP4 to inhibit macrophage expression of TNF, in a manner that is at least partially dependent on both DUSP1 and the modulation of TTP’s phosphorylation state. Despite being an authentic TTP target, IL-6 escapes negative regulation by PGE_2_. The influence of PGE_2_ on *Dusp1* gene expression creates a potent mechanism for context-dependent and gene-specific modulation of inflammatory responses.

## Results

### *Ptgs2* gene expression is negatively regulated by TTP

**Figure 1:**
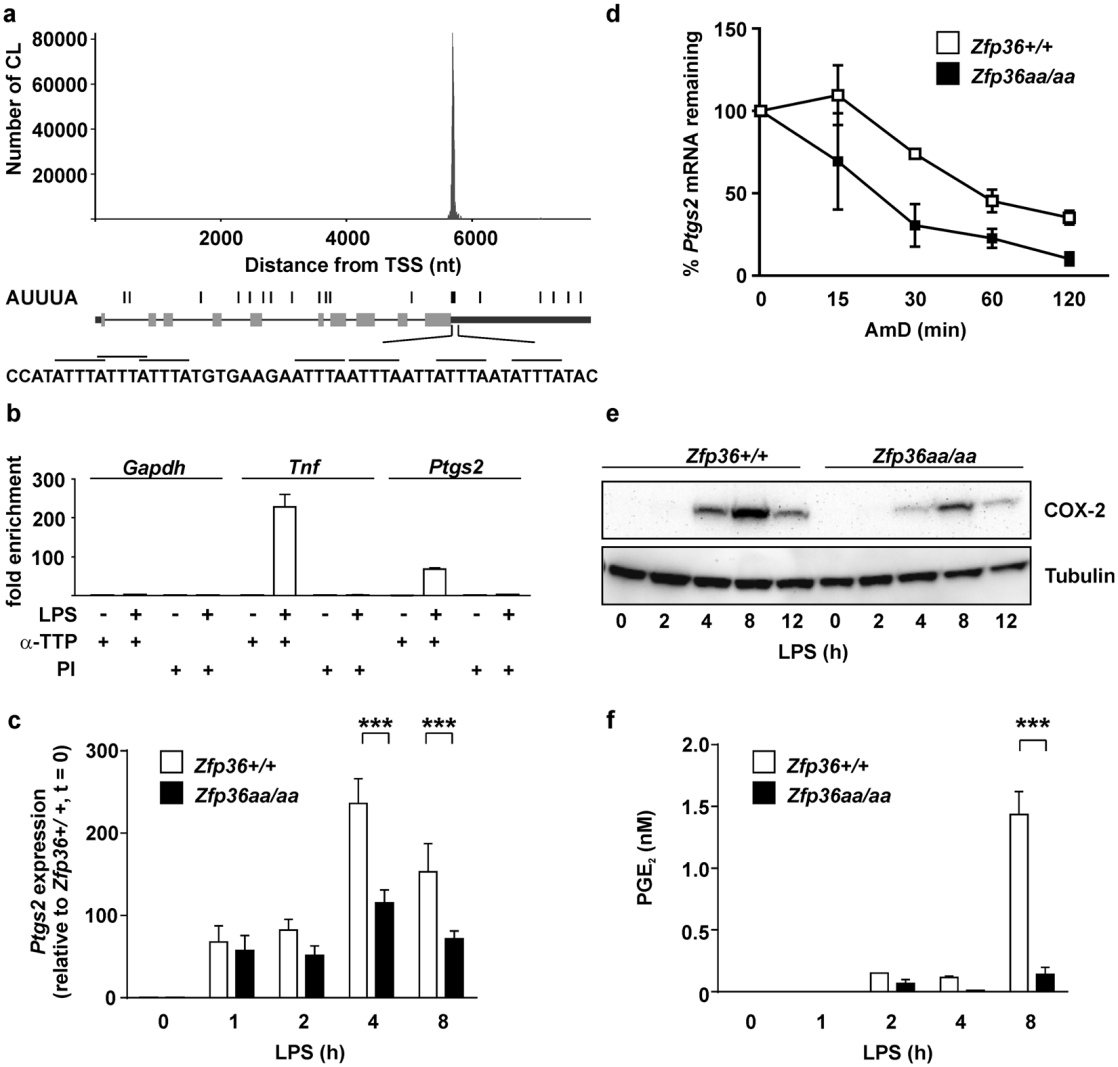
*Ptgs2* gene expression is negatively regulated by tristetraprolin. (**a**) Number of TTP crosslinks (CL) is plotted against position on the *Ptgs2* primary transcript. The primary transcript is illustrated below the graph, with coding exonic sequences as grey bars and non-coding exonic sequences as black bars. Positions of AUUUA motifs are represented above the transcript, and the sequence immediately 3′ to the stop codon is expanded below. Figure adapted from TTP-atlas data (http://ttp-atlas.univie.ac.at)^35^. (**b**) RNA immunoprecipitation was performed on whole cell lysates of RAW264.7 cells, untreated or stimulated with LPS for 2 h, using a TTP antiserum or pre-immune control (PI). *Gapdh*, *Tnf* and *Ptgs2* mRNAs were measured by quantitiative PCR, and fold enrichment was calculated relative to the PI control. A representative of three similar experiments is shown. Error bars indicate SD of triplicate measurements. (**c**) *Zfp36*+/+ and *Zfp36aa*/*aa* BMMs were stimulated with 10 ng/ml LPS for the times indicated, and *Ptgs2* mRNA was measured by qPCR. The graph represents mean ± SEM of three independent BMM cultures of each genotype. ***p < 0.005; Holm-Sidak method for multiple comparison. (**d**) *Zfp36*+/+ and *Zfp36aa*/*aa* BMMs were stimulated with 10 ng/ml LPS for 4 h then actinomycin D was added and *Ptgs2* mRNA measured by qPCR at the intervals indicated. The graph shows mean ± SEM of three independent BMM cultures of each genotype. (**e**) *Zfp36*+/+ and *Zfp36aa*/*aa* BMMs were stimulated with LPS for the times indicated and COX-2 protein was detected by western blotting. Representative of three repeats. (**f**) *Zfp36*+/+ and *Zfp36aa*/*aa* BMMs were stimulated with LPS for the times indicated and PGE_2_ in the tissue culture supernatant was measured by ELISA. The graph represents mean ± SEM of three independent BMM cultures of each genotype. ***p < 0.005; Holm-Sidak method for multiple comparison.

A previous publication described high resolution mapping of TTP binding sites in the mouse macrophage transcriptome^35^. Publicly accessible data from this study (http://ttp-atlas.univie.ac.at) revealed strong binding of TTP to the 3′ UTR of *Ptgs2* mRNA, which was restricted to a region immediately downstream of the open reading frame, containing a cluster of six AUUUA motifs (Fig. 1a). This region mediated regulation of *Ptgs2* mRNA stability by the MAPK p38 signaling pathway, and was recognized by TTP *in vitro* ^24, 25^. Other putative TTP binding sites in the *Ptgs2* 3′ UTR^42^ appear to be recognized by TTP poorly or not at all in mouse macrophages. RNA immunoprecipitation experiments confirmed binding of TTP to *Ptgs2* mRNA in the mouse macrophage cell line RAW264.7 (Fig. 1b). We recently used homologous recombination to generate a novel mouse strain (known as *Zfp36aa*/*aa*), in which serines 52 and 178 of endogenous TTP protein were substituted by non-phosphorylatable alanine residues^43^. The mutant form of TTP could not be inactivated by MK2-mediated phosphorylation, and functioned as a constitutive mRNA destabilizing factor, decreasing the expression of several inflammatory mediators *in vitro* and *in vivo*. *Zfp36aa*/*aa* mice were strongly resistant to experimental endotoxemia and arthritis^43, 44^. *Zfp36aa*/*aa* bone marrow macrophages (BMMs) under-expressed *Ptgs2* mRNA at a steady-state level (Fig. 1c), particularly at later time points. The decrease in steady-state *Ptgs2* expression was accompanied by an increase in its rate of degradation (Fig. 1d). COX-2 protein was expressed at low levels in LPS-treated *Zfp36aa*/*aa* BMMs (Fig. 1e). The LPS-induced release of PGE_2_ was significantly diminished in *Zfp36aa*/*aa* BMMs (Fig. 1f). Heterozygous *Zfp36*+/*aa* BMMs also under-expressed COX-2 protein (Supplemental Figure 1), consistent with our previous description of the non-phosphorylatable TTP mutant as a dominant inhibitor of inflammatory gene expression^43^. These findings confirm that *Ptgs2* mRNA is an authentic, direct target of negative regulation by TTP.

### DUSP1 regulates *Ptgs2* gene expression by modulating TTP phosphorylation

**Figure 2:**
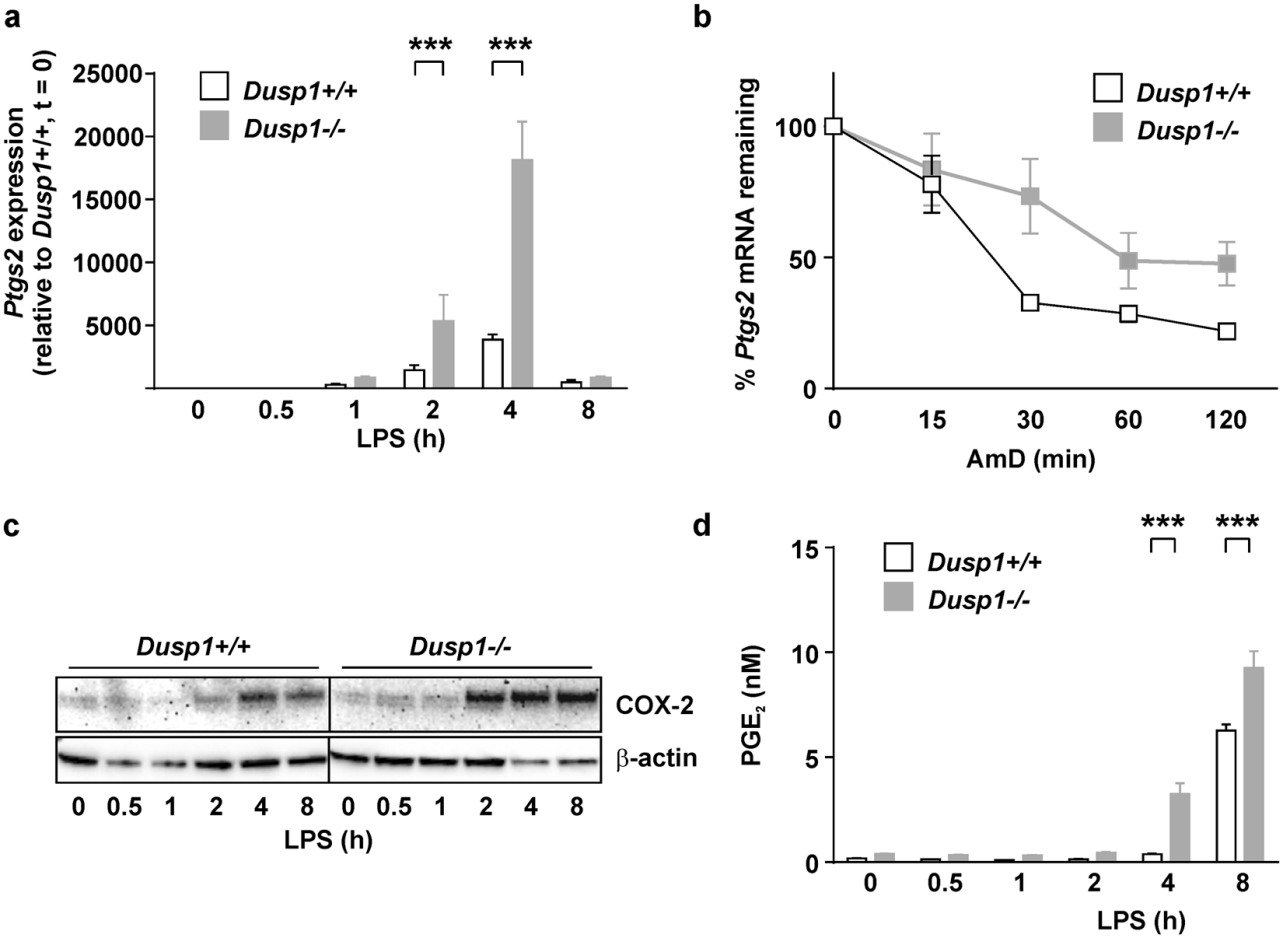
DUSP1 negatively regulates Ptgs2 gene expression at the post-transcriptional level. (**a**) *Dusp1*+/+ and *Dusp1*−/− BMMs were stimulated with 10 ng/ml LPS for the times indicated and *Ptgs2* mRNA was measured by qPCR. The graph represents mean ± SEM of three independent BMM cultures of each genotype. ***p < 0.005; Holm-Sidak method for multiple comparison. (**b**) *Dusp1*+/+ and *Dusp1*−/− BMMs were stimulated with LPS for 4 h then actinomycin D was added and *Ptgs2* mRNA measured by qPCR at the intervals indicated. The graph shows mean ± SEM of three independent BMM cultures of each genotype. (**c**) *Dusp1*+/+ and *Dusp1*−/− BMMs were stimulated with LPS for the times indicated and COX-2 protein was detected by western blotting. Representative of three similar experiments. (**d**) *Dusp1*+/+ and *Dusp1*−/− BMMs were stimulated with LPS for the times indicated and PGE_2_ in the tissue culture supernatant was measured by ELISA. The graph represents mean ± SEM of three independent BMM cultures of each genotype. ***p < 0.005; Holm-Sidak method for multiple comparison.

**Figure 3:**
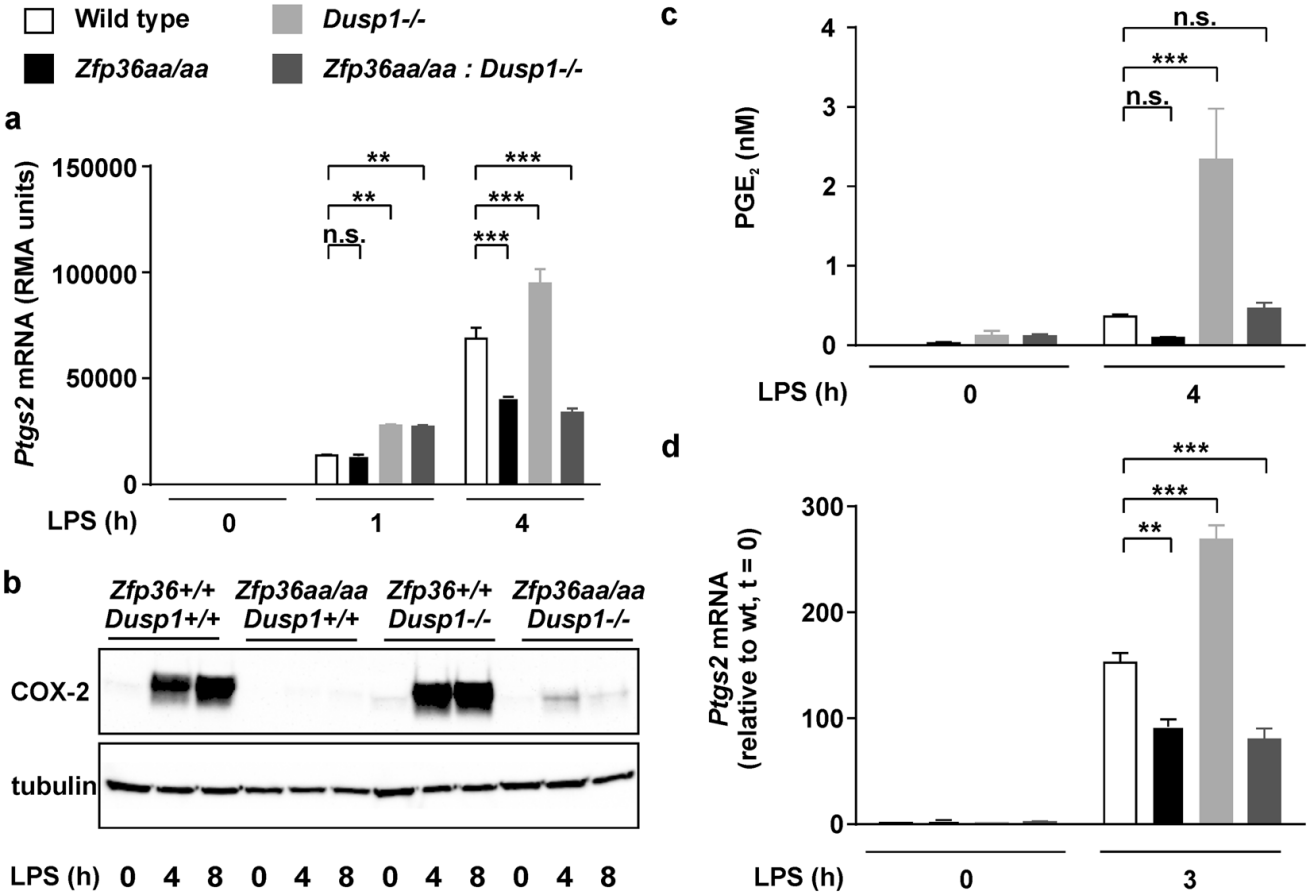
DUSP1 regulates *Ptgs2* gene expression via the modulation of TTP phosphorylation. (**a**) Wild type, *Zfp36aa*/*aa*, *Dusp1*−/− and *Zfp36aa*/*aa: Dusp1*−/− BMMs were treated with 10 ng/ml LPS for 0, 1 or 4 h, and *Ptgs2* mRNA was quantified by microarray. The graph shows RMA (robust multi-array average) units ± SEM from three independent BMM cultures of each genotype. The array was described and extensively validated elsewhere^37^. n.s., not statistically significant. ***p < 0.005, ANOVA. (**b**) BMMs of the same four genotypes were stimulated with LPS for 0, 4 or 8 h, and COX-2 protein was detected by western blotting. Representative of two repeat experiments. (**c**) BMMs of the same four genotypes were stimulated with LPS for 0 or 4 h and PGE_2_ in tissue culture supernatants was measured by ELISA. The graph represents mean ± SEM of three independent BMM cultures of each genotype. n.s., not statistically significant; ***p < 0.005, ANOVA. (**c**) Mice of the same four genotypes were injected intraperitoneally with PBS (n = 2 of each genotype) or 5 mg/kg LPS (n = 4 of each genotype). After 3 h spleens were excised, mRNA isolated, and *Ptgs2* mRNA quantified by RT-PCR with normalization first against *B2m* RNA then against PBS-treated control (*Dusp1*+/+: *Zfp36*+/+) control mice. **p < 0.01; ***p < 0.005, ANOVA.

Steady state levels of *Ptgs2* mRNA were elevated in LPS-treated *Dusp1*−/− BMMs compared with identically treated *Dusp1*+/+ controls (Fig. 2a), accompanied by an increase in *Ptgs2* mRNA stability (Fig. 2b). The expression of COX-2 protein was increased in LPS-treated *Dusp1*−/− BMMs (Fig. 2c), as was the release of PGE_2_ (Fig. 2d). We hypothesized that deletion of the *Dusp1* gene increases the expression of *Ptgs2* via enhanced phosphorylation and inactivation of TTP. To test this hypothesis, expression of *Ptgs2* mRNA, COX-2 protein and PGE_2_ was compared in wild type, *Dusp1*−/−, *Zfp36aa*/*aa* and *Dusp1*−/−: *Zfp36aa*/*aa* BMMs. At 1 h, *Dusp1* gene disruption increased the expression of *Ptgs2* mRNA, but targeted mutation of the *Zfp36* locus was without effect (Fig. 3a). At 4 h, *Ptgs2* mRNA continued to be over-expressed by *Dusp1*−/− BMMs, but in contrast was under-expressed by *Zfp36aa*/*aa* BMMs. Most importantly, *Dusp1*−/−: *Zfp36aa*/*aa* BMMs also under-expressed *Ptgs2* mRNA at 4 h. A similar pattern was observed at the level of COX-2 protein (Fig. 3b) and PGE_2_ biosynthesis (Fig. 3c). To investigate whether the same mechanism was relevant *in vivo*, mice of the same four genotypes were challenged by intraperitoneal injection of LPS, and three hours later *Ptgs2* mRNA expression was measured in spleen, an organ which plays a critical role in systemic responses to endotoxin (Fig. 3c). The LPS challenge strongly enhanced splenic *Ptgs2* expression in mice of all four genotypes. Higher *Ptgs2* expression was seen in *Dusp1*−/− than in wild type control mice, but significantly lower expression was seen in both *Zfp36aa*/*aa* and *Dusp1*−/−: *Zfp36aa*/*aa* mice. Therefore, both *in vitro* and *in vivo*, disruption of the *Dusp1* gene and dysregulation of MAPK signaling enhances *Ptgs2* gene expression in a manner dependent on the phosphorylation of TTP at serines 52 and 178.

### PGE_2_ modulates the phosphorylation of MAPK p38, the expression of DUSP1, COX-2, TNF and IL-6

**Figure 4:**
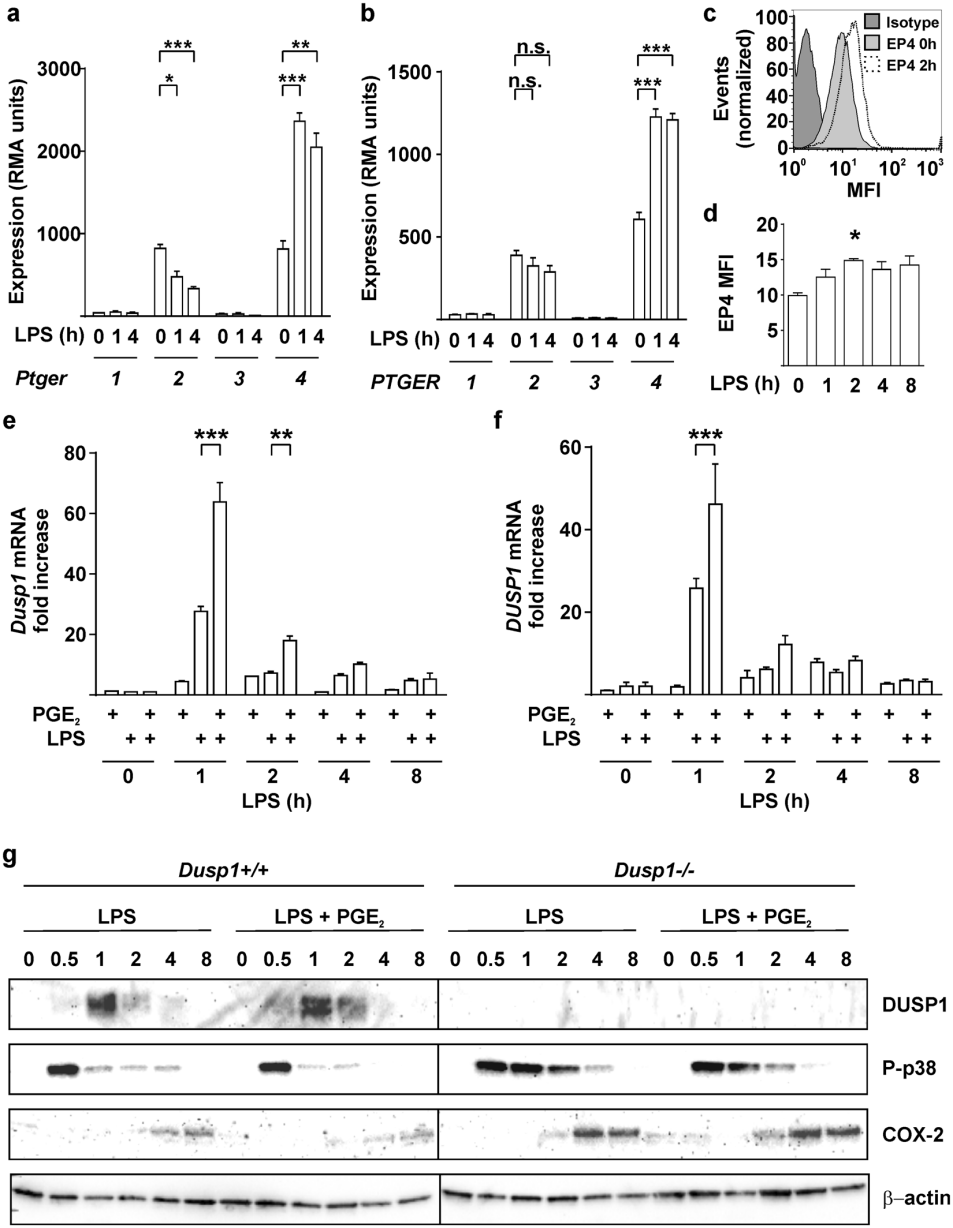
Exogenous PGE_2_ modulates *Dusp1* gene expression and MAPK p38 signaling. Expression of the mouse prostaglandin receptor genes *Ptger1–4* (**a**) or the corresponding human genes *PTGER 1–4* (**b**) was measured by microarray as described in Fig. 3. Graphs represent RMA ± SEM from three independent mouse or human macrophage cultures. n.s., not significant; *p < 0.05; **p < 0.01; ***p < 0.005; ANOVA. (**c**) Representative flow cytometry of EP4 expression in BMMs treated with LPS for 0 or 4 h. (**d**) MFI of EP4 expression was measured at the indicated times after stimulation of BMMs with 10 ng/ml LPS. The graph represents mean ± SEM of three independent wild type BMM cultures. *p < 0.05; ANOVA. (**e**) BMMs were treated for the indicated times with LPS (10 ng/ml), PGE_2_ (1 nM) or both. *Dusp1* mRNA was measured by qPCR, and plotted as fold increase compared to untreated controls. The graph shows mean fold increase ± SEM from three independent BMM cultures. **p < 0.01; ***p < 0.005; ANOVA. (**f**) Primary human monocyte-derived macrophages were treated, and *DUSP1* mRNA was measured, as in (**e**). ***p < 0.005; ANOVA. (**g**) *Dusp1*+/+ and *Dusp1*−/− BMMs were treated with LPS (10 ng/ml) with or without PGE_2_ (1 nM) for the times indicated. DUSP1, phospho-p38, COX-2 and β-actin proteins were detected by western blotting. Representative of four experimental repeats.

We have performed two independent microarray experiments in primary mouse BMMs and one in primary human monocyte-derived macrophages, investigating LPS-induced changes of gene expression. The first mouse microarray experiment has been described^37^ and deposited at the Gene Expression Omnibus (GSE68449), whilst the others are being prepared for submission. All three arrays have been extensively validated. According to all three experiments, both mouse and human primary macrophages expressed mRNAs encoding the prostaglandin receptors EP2 and EP4, whereas mRNAs encoding the other two members of this receptor family, EP1 and EP3, were essentially undetectable (Fig. 4a,b). In macrophages of both species, expression of *Ptger4*/*PTGER4* mRNA was increased by LPS, whereas expression of *Ptger2*/*PTGER2* mRNA was decreased or unaffected. Expression of EP4 in BMMs was confirmed by flow cytometry (Fig. 4c), whilst EP2 protein could not be detected using available reagents. LPS induced a modest increase of EP4 protein levels, which was statistically significant at 2 h (Fig. 4c,d).

Although PGE_2_ is commonly thought of as a pro-inflammatory signaling molecule^2^, it is also known to exert anti-inflammatory effects in myeloid and other cells via activation of the cAMP pathway by EP2 and/or EP4^6, 16, 17, 45^. Expression of the *Dusp1* gene is regulated by cAMP via CREB binding sites in the proximal promoter^46–50^. Induction of DUSP1 expression by PGE_2_ was recently demonstrated in airway smooth muscle cells^51^. We therefore investigated whether PGE_2_ could modulate the expression of DUSP1 and the activity of MAPK signaling pathways in BMMs. On its own, PGE_2_ weakly increased the expression of *Dusp1* mRNA in BMMs (Fig. 4e), although we were not able to detect PGE_2_-induced DUSP1 protein or changes in MAPK p38 phosphorylation. LPS rapidly and transiently increased *Dusp1* mRNA. Combined treatment with LPS and PGE_2_ resulted in cooperative enhancement of *Dusp1* mRNA levels, particularly at the peak of expression, 1 h after the stimulus. Very similar cooperative regulation of *DUSP1* mRNA by LPS and PGE_2_ was observed in primary human monocyte-derived macrophages (Fig. 4f).

In *Dusp1*+/+ BMMs the LPS-induced expression of DUSP1 protein was transient, but was sustained by addition of PGE_2_ (Fig. 4g). The specificity of the antibody is confirmed by the failure to detect corresponding bands in *Dusp1*−/− BMMs. In parallel with prolonged expression of DUSP1, the inactivation of MAPK p38 was accelerated by PGE_2_. In three independent experiments PGE_2_ decreased LPS-induced MAPK p38 phosphorylation by a factor of 0.48 ± 0.06 and increased DUSP1 levels by a factor of 1.80 ± 0.26 (mean fold changes ± SEM) at the 2 h time point. At the 4 h time point phosphorylated MAPK p38 was readily detected in BMMs treated with LPS alone but not in those treated with LPS + PGE_2_. LPS-induced expression of COX-2 protein was also inhibited by addition of PGE_2_. In *Dusp1*−/− BMMs, the activation of MAPK p38 in response to LPS was prolonged and the expression of COX-2 protein was enhanced. Neither of these responses was affected by addition of PGE_2_. Hence the expression of COX-2 is regulated by a negative feedback loop that is mediated by its major catalytic product PGE_2_ and dependent on the expression of DUSP1.

**Figure 5:**
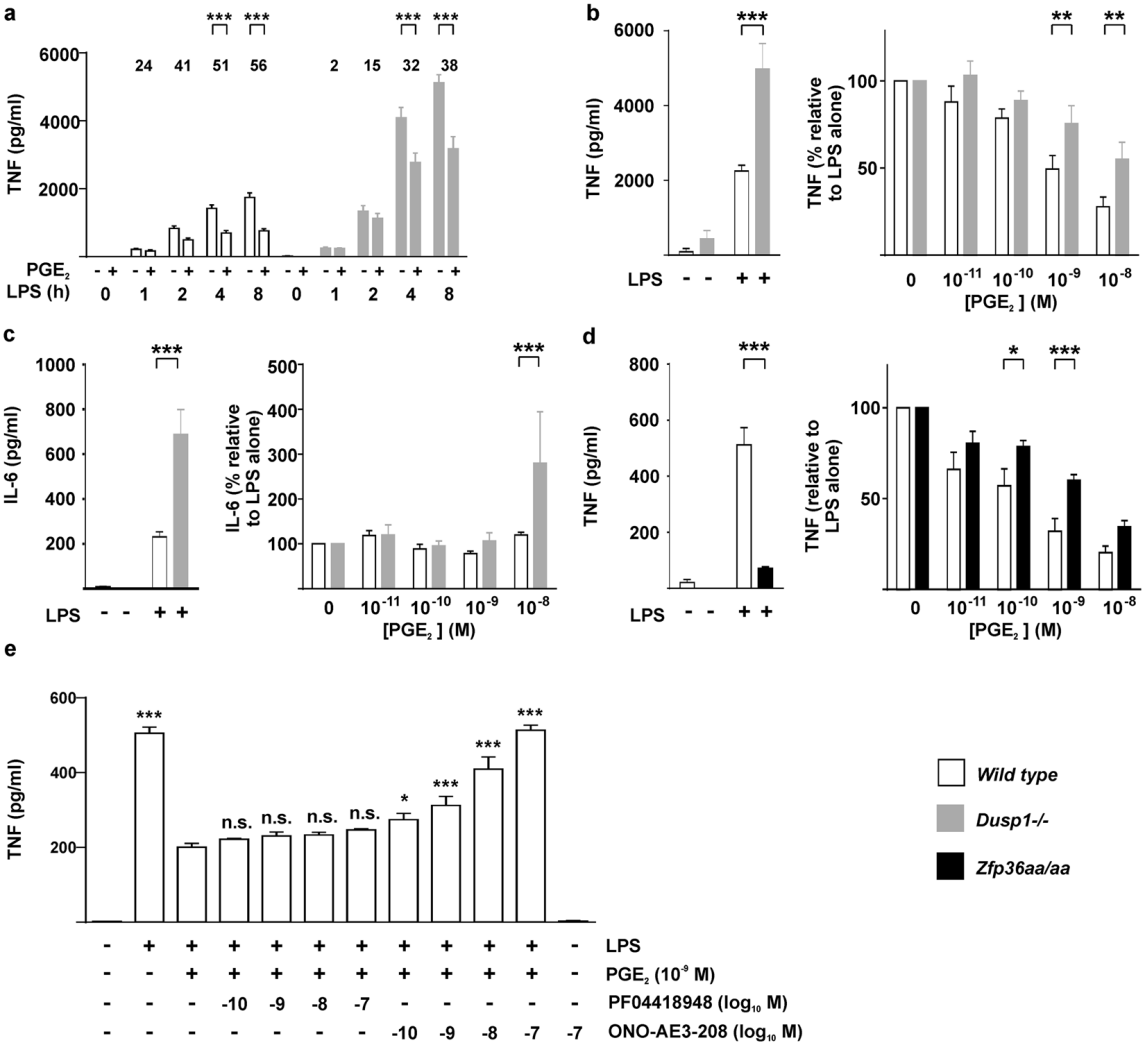
Anti-inflammatory effects of PGE_2_ are gene-selective and partially dependent on DUSP1. (**a**) *Dusp1*+/+ and *Dusp1*−/− BMMs were treated with 10 ng/ml LPS for the indicated times in the absence or presence of 1 nM PGE_2_, and TNF was measured by ELISA. The graph shows mean ± SEM of three independent BMM cultures of each genotype. The numbers above the graph indicate percentage inhibition of TNF release by PGE_2_. ***p < 0.005; Holm-Sidak method for multiple comparison. (**b**) *Dusp1*+/+ and *Dusp1*−/− BMMs were treated with 10 ng/ml LPS for 4 h in the presence of increasing concentrations of PGE_2_, and TNF was measured by ELISA. The graph on the left illustrates absolute quantities of TNF. In the right hand graph, the quantity of TNF in the absence of PGE_2_ is normalized to 100% for each genotype to illustrate differences in the effects of PGE_2_. Mean absolute or relative quantities of TNF from six independent BMM cultures of each genotype are plotted, ±SEM. **p < 0.01; ***p < 0.005; Holm-Sidak method for multiple comparison. (**c**) As (**b**), except that IL-6 was measured. ***p < 0.005. (**d**) As (**b**), except that the comparison was between *Zfp36*+/+ and Zfp36aa/aa BMMs, and n = 3. *p < 0.05; ***p < 0.005; Holm-Sidak method for multiple comparison. (**e**) Wild type BMMs were treated with 10 ng/ml LPS in the absence or presence of 1 nM PGE_2_ and the indicated concentrations of PF04418948 ((**a**) specific EP2 antagonist) or ONO-SE3-208 (**a**) specific EP4 antagonist). TNF was measured by ELISA. The graph represents mean TNF concentration ± SEM from three independent BMM cultures. All statistical comparisons are against the third column, in which BMMs received both LPS and PGE_2_. n.s., not statistically significant; *p < 0.05; ***p < 0.005; ANOVA.

In both *Dusp1*+/+ and *Dusp1*−/− BMMs, exogenous PGE_2_ inhibited the expression of TNF (Fig. 5a). These inhibitory effects were statistically significant at 4 and 8 h after the addition of LPS, and greater in magnitude in *Dusp1*+/+ than *Dusp1*−/− BMMs. Differential sensitivity of *Dusp1*+/+ and *Dusp1*−/− BMMs to anti-inflammatory effects of PGE_2_ was confirmed in dose-response experiments (Fig. 5b). The anti-inflammatory effect was selective, since the expression of IL-6 was not decreased by PGE_2_ in *Dusp1*+/+ macrophages (Fig. 5c). In *Dusp1*−/− BMMs, IL-6 expression was increased by the highest concentration of PGE_2_. *Zfp36aa*/*aa* BMMs were relatively insensitive to inhibitory effects of PGE_2_ on the release of TNF (Fig. 5d), confirming that PGE_2_ modulates TNF expression in part by influencing the phosphorylation of TTP. A selective antagonist of EP2 did not influence the inhibition of TNF biosynthesis by PGE_2_ (Fig. 5e, columns 4–7). A selective EP4 antagonist dose-dependently reversed the suppression of TNF expression by exogenous PGE_2_ (Fig. 5e, columns 8–11) but did not, on its own, increase TNF production (Fig. 5e, column 12). This suggests that EP4 is the major mediator of the anti-inflammatory effects of PGE_2_ in this context.

IL-10 is a potent inhibitor of TNF^52^, and was previously shown to be induced by PGE_2_^18, 53, 54^. We therefore considered whether anti-inflammatory effects of PGE_2_ could be mediated by increased expression of IL-10. Although PGE_2_ increased IL-10 levels, in our hands the effect was modest in magnitude, and achieved statistical significance only in *Dusp1*−/− BMMs (Supplemental Figure 2). *Dusp1*−/− BMMs strongly over-expressed IL-10, but also over-expressed TNF. They also demonstrated increased IL-10 expression in response to PGE_2_, but were relatively insensitive to inhibitory effects of PGE_2_ on TNF release. Therefore, TNF release appears not to be influenced by variations in endogenous IL-10 levels, possibly because of the different kinetics of expression of the two genes. As others have concluded^18, 19^, IL-10 cannot account for anti-inflammatory actions of PGE_2_ in this context.

### The inhibitory effect of exogenous PGE_2_ is strongly time-dependent

**Figure 6:**
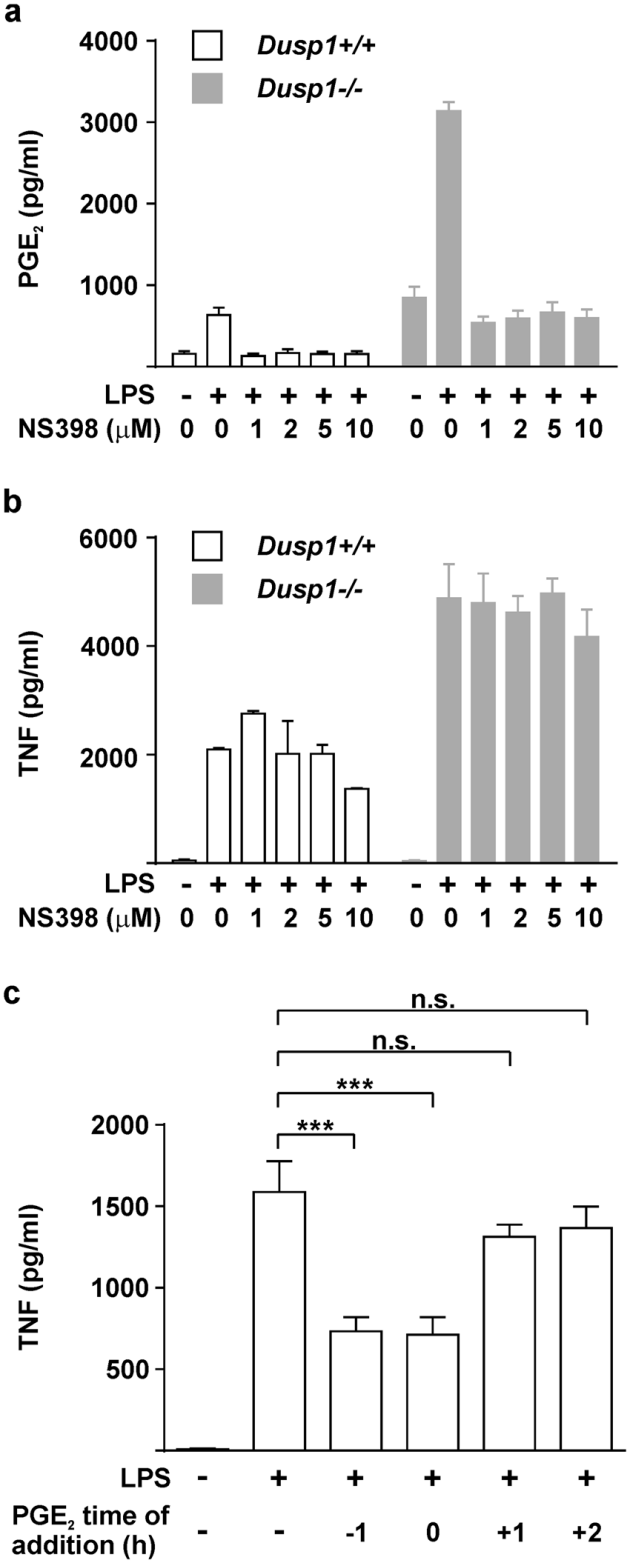
Anti-inflammatory effects of PGE_2_ are strongly time-limited. *Dusp1*+/+ and *Dusp1*−/− BMMs were stimulated with 10 ng/ml LPS in the presence of the indicated concentrations of NS398. PGE_2_ (**a**) and TNF (**b**) were measured by ELISA. Graphs represent mean ± SEM from three independent BMM cultures of each genotype. (**c)** Wild type BMMs were stimulated with 10 ng/ml and harvested four hours later for measurement of TNF by ELISA. PGE_2_ (1 nM) was added at different time points with respect to the addition of LPS at t = 0. The graph shows mean ± SEM from three independent cultures of BMMs. n.s., not statistically significant; ***p < 0.005; ANOVA.

If LPS-induced secretion of PGE_2_ exerts negative feedback via EP4 to limit the expression of inflammatory mediators, the inhibition of COX-2 function and endogenous PGE_2_ synthesis would be expected to increase TNF release. To test this, we first confirmed that LPS-induced release of PGE_2_ was effectively eliminated by a selective inhibitor of COX-2 enzymatic activity, NS398 (Fig. 6a). The effect of NS398 on LPS-induced release of TNF was then tested in *Dusp1*−/− and *Dusp1*+/+ BMMs. Neither genotype of BMM displayed any change of TNF expression in the presence of a concentration of NS398 sufficient to inhibit LPS-induced PGE_2_ release (Fig. 6b). Differences of timing could explain why the expression of TNF is sensitive to exogenously added but insensitive to endogenously produced PGE_2_. The accumulation of PGE_2_ is a gradual process requiring both *de novo* gene expression and several catalytic steps, such that PGE_2_ levels in cell culture medium become elevated only after 4 h (Figs 1f and 2d). In contrast the activation of *Dusp1* gene expression is rapid and transient, and cooperative regulation by LPS and PGE_2_ is short-lived (Fig. 4e). Therefore, in cells exposed to synchronous stimulation, most of the important regulatory processes are likely to have occurred before endogenously generated PGE_2_ levels are high enough to have an impact. To test this concept, BMMs were stimulated for 4 h with LPS, without addition of exogenous PGE_2_, or with addition 1 h before the LPS stimulus, at the same time as the stimulus, 1 h or 2 h after the stimulus. PGE_2_ was able to inhibit TNF biosynthesis only if added before or at the same time as the LPS stimulus (Fig. 6c). Although the majority of LPS-induced TNF production occurred after 1 h (Fig. 5a), addition of PGE_2_ at 1 h or later had no significant inhibitory effect.

## Discussion

At early time points in the response to LPS, expression of the *Ptgs2* gene was enhanced by *Dusp1* deletion but unaffected by gain of function mutation of *Zfp36* (Fig. 3a, 1 hour). This suggests the existence of a mechanism of regulation of *Ptgs2* expression by MAPKs, independent of TTP phosphorylation. For example it is possible that enhanced early MAPK p38 activity in *Dusp1*−/− BMMs contributes to transcriptional activation of the *Ptgs2* gene via NF-κB or other transcription factors^55^. However, it is clear that this mechanism dwindles in importance as the time course progresses and the contribution of the DUSP1-TTP axis dominates the expression of *Ptgs2* mRNA both *in vitro* (Fig. 3a, 4 hour) and *in vivo* (Fig. 3d), the cumulative expression of COX-2 protein (Fig. 3b) and the production of PGE_2_ (Fig. 3c). We could find no evidence that the targeted mutation of TTP had any impact on NF-κB activity or transcription of the target genes that we examined^43^. In contrast both deletion of the *Dusp1* gene and targeted mutation of the *Zfp36* gene clearly influenced the stability of *Ptgs2* mRNA (Figs 1d and 2b), as well as that of other TTP targets^37, 43^. These findings place the *Ptgs2* gene alongside *Tnf*, *Cxcl1*, *Cxcl2*, *Ifnb1*, *Il1b* and several others, as targets of the DUSP1-TTP regulatory axis, which tightly couples mRNA stability to the activity of the MAPK p38 signaling pathway^30, 34, 35, 37, 43, 56^. Increased expression of the *Ptgs2* gene may contribute to the innate immune pathology of both *Dusp1*−/− and *Zfp36*−/− mice^27, 38^, whilst decreased expression of *Ptgs2* may play a role in the resistance of *Zfp36aa*/*aa* to experimental endotoxemia and arthritis^43, 44^.

**Figure 7:**
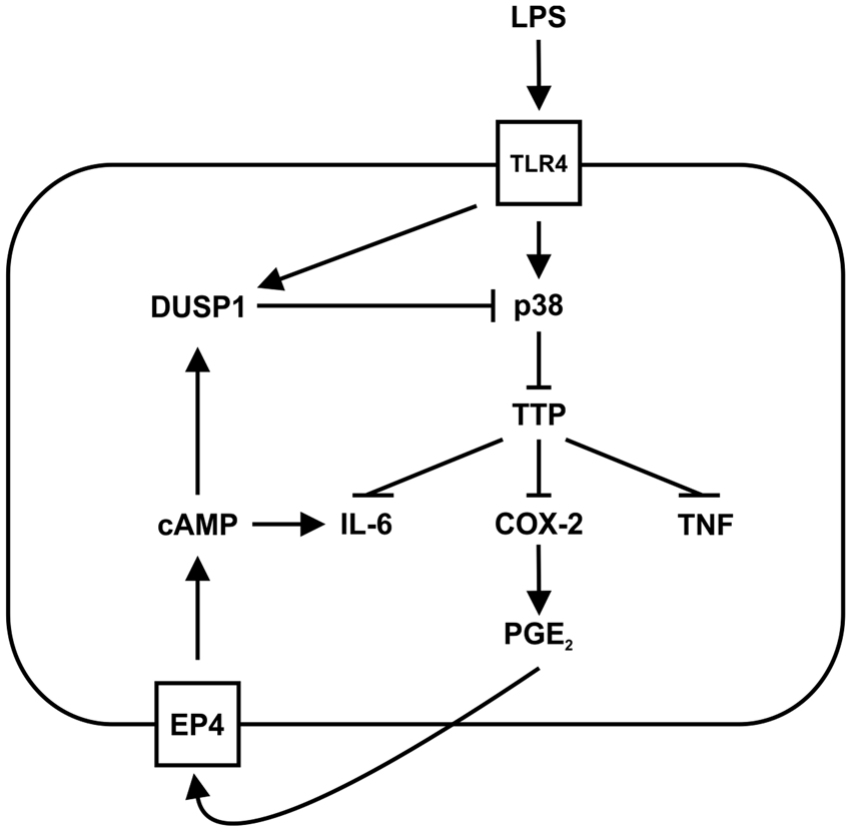
Schematic representation of the feedback control of macrophages by PGE_2_, DUSP1 and TTP. Note that the effects of PGE_2_-mediated feedback are delayed because of the gradual accumulation of PGE_2_, and therefore operate in a paracrine rather than autocrine fashion. The figure ilustrates possible regulatory connections, and is not intended to capture all the temporal complexities of feedback control.

The second half of our study focuses on possible consequences of altered PGE_2_ release by LPS-activated macrophages. Both mouse and human primary macrophages expressed the adenylyl cyclase-coupled PGE_2_ receptors EP2 and EP4 and displayed cooperative regulation of *Dusp1*/*DUSP1* gene expression by LPS and exogenous PGE_2_. In wild type murine macrophages, exogenous PGE_2_ accelerated the decline of MAPK p38 activity and reduced the expression of COX-2 protein. In *Dusp1*−/− macrophages neither of these effects was seen (Fig. 4). Our findings reveal a negative feedback loop that is illustrated schematically in Fig. 7. PGE_2_ down-regulates the expression of COX-2 by increasing the expression of DUSP1, decreasing the activity of MAPK p38 and enhancing the function of TTP. This contrasts with the MAPK p38-dependent positive feedback regulation of COX-2 expression by PGE_2_ that has been described by others^45, 57, 58^. We found no evidence for activation of MAPK p38 or induction of COX-2 expression by PGE_2_ in primary murine macrophages. The reason for the discrepancy is not clear.

Exogenous PGE_2_ strongly inhibited the LPS-induced expression of TNF by primary mouse macrophages (Fig. 5). The PGE_2_ receptor EP4 contributed to this anti-inflammatory effect, whereas EP1 or EP3 appeared not to be expressed, and we could not find evidence for involvement of EP2. We are currently investigating whether LPS-induced up-regulation of EP4 sensitizes macrophages to anti-inflammatory effects of PGE_2_. Importantly, *Dusp1* gene disruption and targeted mutation of the *Zfp36* (TTP) gene had opposite effects on the expression of TNF, increasing or decreasing it, respectively (Fig. 5b and d, left panels). However, both genetic modifications rendered macrophages similarly insensitive to PGE_2_-mediated suppression of TNF synthesis (Fig. 5b and d, right panels). These results establish a detailed, novel molecular mechanism for anti-inflammatory effects of PGE_2_.

IL-10 is a potent anti-inflammatory cytokine^52, 59^. IL-6 is not merely pro-inflammatory, but also has important functions in resolution and tissue remodeling^60^. Both *Il6* and *Il10* genes are well-documented TTP targets^27^, yet they escaped negative regulation by PGE_2_. In fact, both were up-regulated by PGE_2_ in macrophages lacking DUSP1 (Figs 5c and 2). Significantly, both genes are positively regulated by cAMP signaling and have been shown to be induced by PGE_2_^18, 54, 61–65^. This may be an adaptation that permits their continued expression under conditions favoring the suppression of TNF. According to this model (illustrated for *Il6* in Fig. 7), PGE_2_ exerts opposing, direct positive and indirect negative effects on gene expression. It positively regulates expression at the transcriptional level via cAMP signaling, and negatively regulates expression post-transcriptionally via induction of DUSP1, inhibition of MAPK p38 and enhancement of TTP function. In wild type macrophages these effects are balanced. In the absence of DUSP1, the negative regulation is lost and the positive regulation unmasked.

Macrophage TNF biosynthesis was inhibited by exogenously added PGE_2_ but insensitive to endogenously produced PGE_2_, most likely due to a time delay in LPS-induced PGE_2_ biosynthesis (Fig. 6). This discrepancy highlights the artificial nature of *in vitro* experiments, in which relatively pure cell populations are exposed to a synchronous stimulus. *In vivo*, as cells migrate to a site of infection or tissue damage, they will encounter a milieu that is continually changing as the inflammatory response peaks and resolves. We suggest that the local biosynthesis of PGE_2_ helps to shape this program by modulating the responses of cells arriving at different stages. A monocyte or macrophage arriving early and encountering LPS in the absence of PGE_2_ will generate a response very different from that of a late-arriving cell that encounters LPS in the presence of high concentrations of PGE_2_. In this sense, PGE_2_ may provide temporal context for responses to pro-inflammatory stimuli, assisting in transitions between initiation and resolution phases. This mode of action of PGE_2_ may help to explain why cyclooxygenase inhibition sometimes does not have straightforwardly anti-inflammatory consequences, or can even be toxic to resolution^11, 12, 66–68^.

## Methods

### Animals and experimental procedures

All mice were maintained at the Biomedical Services Unit of the University of Birmingham. Animal care and experimental procedures were performed according to Home Office gidelines under PPL 40/8003, and approved by the University of Birmingham Local Ethical Review Committee.

*In vivo* LPS challenge was performed by intraperitoneal injection of PBS or 5 mg/kg LPS. After three hours all mice were humanely killed. Spleens were recovered and homogenized in RLT buffer (Qiagen) prior to isolation of RNA as described below.

### Reagents

LPS (*E. coli* EH100) was from Enzo Life Sciences. PGE_2_, PF04418948 and ONO-AE3-208 were from Cayman Chemical. Antibodies used in western blotting were from Santa Cruz (COX-2, sc-1745; DUSP1, sc-373841), Cell Signaling Technology (phosphorylated MAPK p38, #9211), Sigma Aldrich (tubulin, T9026 and β-actin, A1978). Flow cytometry antibodies against EP2 and EP4 were from Cayman Chemical (16684 and 16625). All other reagents were from Sigma Aldrich. The generation of *Dusp1*−/−, *Zfp36aa*/*aa* and *Dusp1*−/−: *Zfp36aa*/*aa* mouse strains was previously described^37^. All strains were back-crossed to C57/BL6J for at least ten generations.

### Cell culture

Mice between 6 and 12 weeks of age were humanely culled, bone marrow flushed from femurs, and bone marrow-derived macrophages (BMMs) obtained by culture for 7 days in RPMI1640 containing 10% heat-inactivated FCS and 100 ng/ml M-CSF. Prior to experimentation, macrophage purity was assessed by flow cytometry. Routinely >95% of cells were F4/80+ at the end of the 7 day culture period. BMMs were harvested by scraping, seeded at a density of 10^6^/ml in appropriate culture vessels and rested overnight in the absence of M-CSF before being stimulated. Primary human macrophages were generated from peripheral blood monocytes of healthy donors as previously described^52^, by culture for 5–7 days in RPMI1640 containing 10% heat-inactivated FCS and 100 ng/ml M-CSF. After this time, cells were harvested by scraping, seeded at a density of 10^6^/ml and rested overnight in the absence of M-CSF before being stimulated.

### Measurement of mRNA

RNA was isolated from primary human or mouse macrophages using QIAshredder columns and RNeasy kits (Qiagen). cDNA was generated using iScript cDNA Synthesis kits (Bio-Rad). Gene expression was measured by quantitative PCR with a Roche Light-Cycler 480 Mark II, using custom-designed primers (Eurofins) and SYBR Premix Ex Taq (Takara). Relative expression was calculated using th ΔΔCt method with *B2m* or *B2M* mRNA for normalization.

For microarray analysis of gene expression in primary mouse macrophages, RNA was prepared as above and processed as described^37^. For microarray analysis of gene expression in human monocyte-derived macrophages, RNA was prepared as above, cleaned and concentrated then applied to Affymetrix HuGene 1.0 ST arrays. Data analysis was essentially as described^37^, using two-way mixed model ANOVA in Partek Genomics Suite version 6.6. One of the two mouse microarray experiments has been submitted to the Gene Expression Omnibus (GSE68449) at the National Center for Biotechnology Information (http://www.ncbi.nlm.nih.gov/). The second mouse BMM microarray and the human monocyte-derived macrophage microarray experiments are more fully described by manuscripts currently in preparation, and the data will be submitted to GEO in full. In the interim, these datasets are available from the author on reasonable request.

### RNA Immunoprecipitation

2 × 10^7^ RAW264.7 cells were left untreated or stimulated with 10 ng/ml LPS for 1 h, then harvested by scraping, washed twice with ice-cold PBS and lysed by repeated freeze-thawing in 1 ml of ice cold polysome lysis buffer (100 mM KCl, 10 mM HEPES [pH 7.0], 5 mM MGCl_2_, 0.5% Nonidet P-40, 1 mM DTT, 100 U/ml RNase inhibitor, protease and phosphatase inhibitor cocktails [Roche]). Immunoprecipitations and mRNA measurements were essentially as described^43^, except that a pre-immune (PI) rabbit serum was used as the immunoprecipitation control, and quantitative PCR was used to derive fold enrichment of various mRNAs in the anti-TTP immunoprecipitates compared with the PI controls (calculated as 2^−ΔCt^).

### Measurement of proteins and PGE_2_

Intracellular proteins were detected by western blotting using reagents listed above. Secreted IL-6, TNF and PGE_2_ were detected by sandwich ELISA using commercial kits, according to manufacturers’ instructions. For detection of cell surface EP4, BMMs were fixed and permeabilized using Cytofix/Cytoperm solution according to manufacturer’s instructions (BD Biosciences) and subjected to flow cytometry using an APC-coupled antibody, Cyan flow cytometer (Beckman Coulter) and FloJo software (TreeStar Inc).

### Statistics

Statistical analysis was performed using GraphPad Prism 6.07. Pairwise comparisons were performed using Holm-Sidak’s method, whilst multiple comparisons were performed using Dunnett’s multiple comparison test. The same marks are used throughout; n.s., not significant; *p < 0.05; **p < 0.01; ***p < 0.005.

## Electronic supplementary material

Supplementary information
