# Supplementary material for: Macrophage responses to lipopolysaccharide are modulated by a feedback loop involving prostaglandin E2, dual specificity phosphatase 1 and tristetraprolin
Source: Sci Rep. 2017 Jun 28;7:4350. doi: 10.1038/s41598-017-04100-1 (PMC5489520; doi:10.1038/s41598-017-04100-1)
Supplement: Supplementary file 1 — Supplementary information [file 41598_2017_4100_MOESM1_ESM.pdf]

Macrophage responses to lipopolysaccharide are modulated by a feedback loop involving prostaglandin E2, dual specificity phosphatase 1 and tristetraprolin.

Tina Tang, Thomas E. Scambler, Tim Smallie, Helen E. Cunliffe, Ewan A. Ross, Dalya R. Rosner, John D. O'Neil and Andrew R. Clark.

Supplementary Figures.

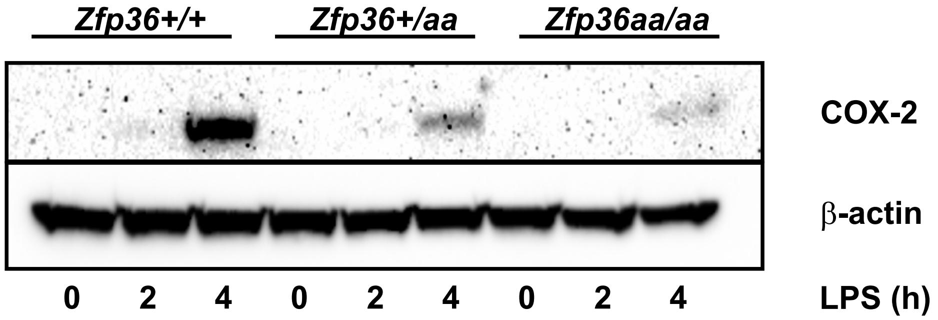

Supplementary Figure 1. COX-2 protein is under-expressed by *Zfp36+/aa* BMMs. *Zfp36+/+*, *Zfp36+/aa* and *Zfp36aa/aa* littermates were identified by a PCR-based screen, BMMs were generated and stimulated for 0, 2 or 4 h with 10 ng/ml LPS. COX-2 and β-actin proteins were detected by western blotting. Representative of two experiments.

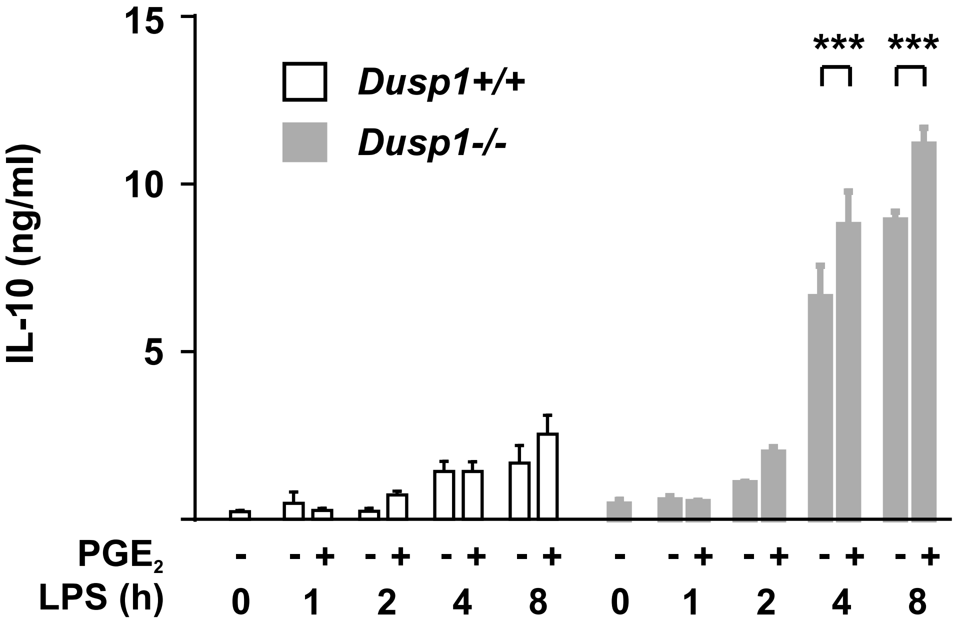

Supplementary Figure 2. Effects of PGE_2 on expression of IL-10. *Dusp1+/+* and *Dusp1-/-* BMMs were treated with 10 ng/ml LPS for the indicated times in the absence or presence of 1 nM PGE_2, and secreted IL-10 was measured by ELISA. The graph shows mean +/- SEM from three independent BMM cultures of each genotype. ***, $p < 0.005$ (t test by Holm-Sidak method).
